# Effects of Radio-Taiso on Health-related Quality of Life in Older Adults With Frailty: a Randomized Controlled Trial

**DOI:** 10.2188/jea.JE20230317

**Published:** 2024-10-05

**Authors:** Yosuke Osuka, Narumi Kojima, Kaori Daimaru, Risa Ono, Masamitsu Sugie, Takuya Omura, Keiko Motokawa, Takuya Ueda, Kazushi Maruo, Toshihiko Aoyama, Shigeru Inoue, Hiroyuki Sasai

**Affiliations:** 1 Research Team for Promoting Independence and Mental Health, Tokyo Metropolitan Institute for Geriatrics and Gerontology, Tokyo, Japan; 2 Department of Frailty Research, Center for Gerontology and Social Science, Research Institute, National Center for Geriatrics and Gerontology, Aichi, Japan; 3 Japan Radio-Taiso Federation, Tokyo, Japan; 4 Research Team for Human Care, Tokyo Metropolitan Institute for Geriatrics and Gerontology, Tokyo, Japan; 5 Department of Endocrinology and Metabolism, Hospital, National Center for Geriatrics and Gerontology, Aichi, Japan; 6 Department of Metabolic Research, Research Institute, National Center for Geriatrics and Gerontology, Aichi, Japan; 7 The Tokyo Metropolitan Support Center for Preventative Long-term and Frail Elderly Care, Tokyo Metropolitan Institute for Geriatrics and Gerontology, Tokyo, Japan; 8 Department of Biostatistics, Institute of Medicine, University of Tsukuba, Ibaraki, Japan; 9 Department of Preventive Medicine and Public Health, Tokyo Medical University, Tokyo, Japan

**Keywords:** exercise, patient-reported outcome, randomized controlled trial, multi-component physical activity

## Abstract

**Background:**

Radio-Taiso, a long-standing exercise program in Japan, could be a sustainable public health strategy for maintaining quality of life (QoL) in older adults with frailty. This study aimed to investigate whether Radio-Taiso provided greater benefits for health-related quality of life (HRQoL) and to identify the mechanisms underlying the effectiveness in this population.

**Methods:**

A 12-week randomized controlled trial enrolled 226 older Japanese adults with pre-frailty or frailty, assessed using the modified frailty phenotype. Participants were randomly allocated to the intervention (Radio-Taiso + nutrition program) or control (nutrition program) groups. The Radio-Taiso program comprised five 60-min group sessions and daily practice at the participants’ homes. The primary outcome was the change in the mental domain of HRQoL, assessed using the SF-36^®^. The secondary outcomes included six physical fitness items and exercise self-efficacy.

**Results:**

Overall, 104 and 105 participants in the intervention and control groups, respectively, were analyzed based on the intention-to-treat principle. The median daily practice rate of Radio-Taiso was 94.1% (interquartile range, 73.2–98.8%). Although general linear models adjusted for baseline values and allocation stratification factors showed that the intervention group obtained greater benefits (adjusted mean differences) in the up-and-go (0.3; 95% confidence interval [CI], 0.1 to 0.6 s), 2-min step-in-place (−3.2; 95% CI, −6.2 to −0.2 steps) tests, and exercise self-efficacy scale (−1.4; 95% CI, −2.6 to −0.1 points) than the control group, there were no group differences in changes in the mental domain score of HRQoL.

**Conclusion:**

Radio-Taiso provided greater benefits for agility/dynamic balance, aerobic endurance, and exercise self-efficacy in older adults with frailty; however, these changes do not improve HRQoL.

## INTRODUCTION

Frailty, a vulnerability of homeostasis to respond appropriately to stressors due to cumulative decline in physiological reserves with aging,^1^ poses risks of adverse health outcomes, including falls and disability.^2^ Identifying older adults with frailty early and connecting them to appropriate interventions is crucial. Previous clinical trials have reported that exercise and/or nutritional interventions help reduce frailty scores; however, the clinical interpretation of how the changes affect the way of feeling in daily lives among older adults with frailty remains unclear.^3^ Clinical trials that focus on patient-reported outcomes, including health-related quality of life (HRQoL), would reinforce a patient-centered approach by evaluating intervention effects based not only on objective measures, such as biological markers, but also on the perspectives of those involved, including changes and feelings in patients’ daily lives. Consequently, such an approach contributes to translating evidence into care that is more effective and appropriate for the patient in practice.^4^

A meta-analysis reported that individuals with frailty had significantly lower HRQoL than those without, showing moderate-to-large standardized mean differences.^5^ Another network meta-analysis on managing frailty suggested exercise interventions as a potentially effective strategy for improving HRQoL in older adults^6^; however, evidence on the effect of home-based exercise interventions on HRQoL remains limited.^7^ Moreover, stakeholders, including frail older adults, their caregivers, and administrators, express concerns that previous interventions have focused primarily on physical aspects. Clinical trials focusing on psychosocial aspects would also be beneficial for addressing the various needs of the parties involved.^3^

**Figure 1.**
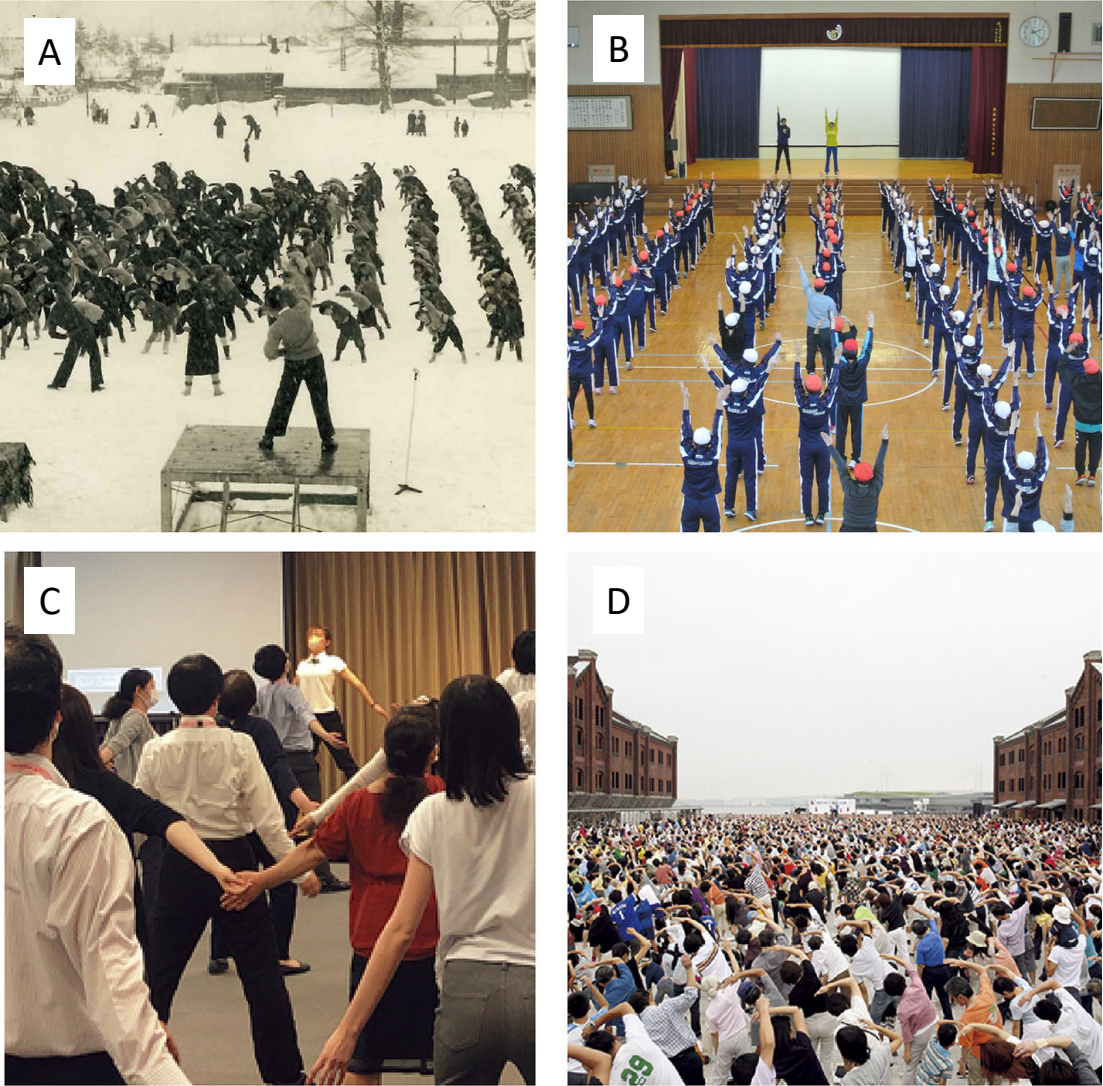
Photographs of the Radio-Taiso program. They were gathering and practicing in school among old and present days (**A** and **B**), during workplace events (**C**), and social events (**D**). All pictures were provided courtesy of Japan Post Insurance Co., Ltd.

Radio-Taiso, “Japan’s National Exercise,” is the most famous and traditional exercise program in Japan. It was developed in 1928 by the Postal Life Insurance Bureau of the Ministry of Communications.^8^ Most older adults are familiar with Radio-Taiso because of its history and integration into various settings, including schools, workplaces, and communities (Figure 1).^9^ Moreover, because Radio-Taiso is broadcasted on public radio and television programs daily by the Japan Broadcasting Corporation, it is available to everyone free of charge. The Radio-Taiso is considered a multi-component exercise program because it incorporates various physical fitness domains, including muscle strength, flexibility, agility, balance, endurance, and coordination, which are required for the smooth execution of movements.^10^ The World Health Organization has recommended that older adults participate in aerobic, muscle-strengthening, and multi-component physical activities to maintain their health.^11^ Radio-Taiso could serve as a sustainable public health strategy for promoting adherence to physical activity guidelines and preventing the progression of frailty in community-dwelling older Japanese adults.

**Figure 2.**
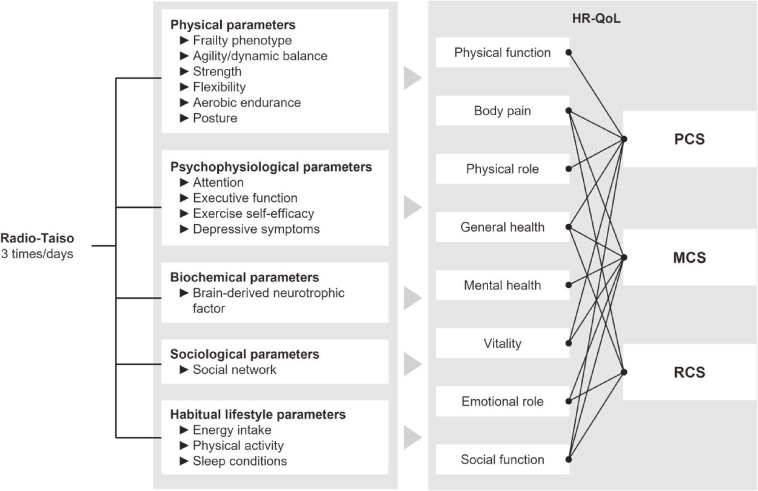
A conceptual model elucidating the mechanisms of the effect of the home-based Radio-Taiso exercise program. HRQoL, health-related quality of life; MCS, mental component summary; PCS, physical component summary; RCS, role/social component summary. Adapted from Osuka et al.^10^ Effects of a home-based Radio-Taiso exercise programme on health-related quality of life in older adults with frailty: protocol for an assessor-blind randomised controlled trial. BMJ Open 22(9): e063201. doi: 10.1136/bmjopen-2022-063201, licensed under Creative Commons Attribution 4.0 Unported License.

While identifying the effectiveness of home-based Radio-Taiso is significant from a public health perspective, the safety, feasibility, and potential effectiveness of performing the exercise at home in a frail population were previously unknown. Our previous phase 2 randomized controlled trial demonstrated that home-based Radio-Taiso is safe and acceptable, providing clinically meaningful benefits for the mental domain of HRQoL in older adults with frailty.^12^ However, the effectiveness and underlying mechanisms of the intervention in the mental domain of HRQoL need validation through a more conclusive clinical trial. The primary objective of this phase 3 trial was to assess whether home-based Radio-Taiso would benefit the mental domain of HRQoL in older adults with frailty. Additionally, the second objective was to exploratorily and comprehensively identify the underlying mechanisms of the effectiveness of home-based Radio-Taiso (Figure 2).^10^

## METHODS

### Design, setting, and ethics

This 12-week, randomized, parallel-designed, two-arm trial was conducted using the CONSORT and CONSORT-PRO extension statements (See eMaterial 1).^13^^,^^14^ The interventions were conducted in participants’ homes, at the Tokyo Metropolitan Institute for Geriatrics and Gerontology (TMIG), or at a community center.

This study was conducted in accordance with the principles embodied in the Declaration of Helsinki. This trial protocol was approved by the Research Ethics Committee of TMIG on December 16, 2021, and published in the University Hospital Medical Information Network Clinical Trials Registry on March 20, 2022 (trial number: UMIN000047229). A detailed trial protocol was submitted as a protocol paper on March 24, 2022.^10^ After these procedures, recruitment was initiated. Written informed consent was obtained from all participants before the baseline assessment.

### Recruitment and eligibility criteria

Participants were recruited from a population-based trial-ready cohort at the TMIG between October 2021 and February 2022. In April 2022, individuals meeting the frailty or pre-frailty criteria of the revised Japanese version of the Cardiovascular Health Study^15^ received an invitation letter for participation and an eligibility criteria checklist. Pre-frailty or frailty was defined based on the presence of 1–2 or ≥3 of the five limitations (slowness, weakness, exhaustion, inactivity, and weight loss) in participants, respectively.^2^^,^^15^ The exclusion criteria are shown in eMaterial 2.^10^

### Randomization and blinding

Randomization and blinding were performed according to a predetermined process.^10^ After the baseline assessment, participants were randomly assigned to the intervention (Radio-Taiso + nutrition program) or control (nutrition program only) groups in a 1:1 ratio based on a pre-generated allocation code. Randomization was performed using computerized block randomization (block size = 2), stratified based on sex (male or female), age (<75 or ≥75 years), and frailty severity (frailty or pre-frailty). The principal investigator (YO) sent the participants’ identification codes to a clinical statistician (KaM), who had no contact with them. An independent staff member received assignment codes from the KaM and assigned participants to groups. The group labels remained undisclosed to assessors or statistical analysts (HS) until the main analysis was completed.

### Intervention

The intervention began on June 20, 2022, and ended on September 11, 2022. The participants were asked not to start any new exercise or nutritional programs during the intervention. A nutritional program was provided for both groups to mitigate the ethical disadvantages that the control group may experience.

### Radio-Taiso

Radio-Taiso comprises the following three exercise programs: Radio-Taiso No. 1 (3 min 10 s), No. 2 (3 min 5 s), and Minna no Taiso (4 min 30 s). Radio-Taiso No. 1, with the longest history, was developed to be familiar to all generations, from children to older adults. The exercise intensity of this exercise program is approximately 3.7 metabolic equivalents.^16^ Radio-Taiso No. 2 has a slightly higher intensity than Radio-Taiso No. 1 and was specifically designed for adults to perform in the workplace. Minna no Taiso, with the most recent history, was designed to be of low intensity, ensuring familiarity for everyone. Each exercise program comprises 8–13 rhythmic and systemic movements performed using music. The objectives of each movement and video URLs are detailed in our previously published protocol.^10^

The intervention group was asked to participate in six 60-min face-to-face sessions with certified instructors from the Japan Radio-Taiso Federation. One week before the start of the intervention period, instructions for performing the three exercise patterns correctly and safely were provided (first session). Review instructions were provided weekly (second to fifth sessions) during the first 4 weeks of the intervention. The last (sixth) session was planned to be conducted 8 weeks after the start of the intervention to review the essentials of each movement; however, it was canceled due to the coronavirus disease 2019 (COVID-19) pandemic.

At home, participants were instructed to perform the three Radio-Taiso patterns once daily while watching a DVD or TV program broadcast by the Japan Broadcasting Corporation and record daily in their exercise diary whether they 1) practiced the three types of Radio-Taiso and 2) complied with the key points for practicing them effectively.

### Nutrition program

One week before the intervention, a registered dietitian presented an overview of the nutritional program to the participants. The detailed nutritional program can be found in our previously published protocol.^10^

### Outcomes

The primary outcome was the change in the Mental Component Summary score of the HRQoL. HRQoL was assessed using the Japanese version of SF-36.^17^^,^^18^ The SF-36 is organized into the following eight subdomains: physical function, physical role, body pain, general health, vitality, social function, emotional role, and mental health, which are aggregated into the Mental, Physical, and Role and Social Component Summary scores.^19^ All scores were indicated as T-scores based on the 2017 Japanese national standards.^20^ Differences of ≥3 points in the Mental Component Summary score could be interpreted as minimal clinically significant differences.^21^

To determine the mechanisms underlying the effects of the primary outcome and their impact on lifestyle, the following markers of the secondary outcomes were assessed^10^: 1) Physical Component Summary score, Role and Social Component Summary score, and eight subscales of the HRQoL; 2) physical markers, including frailty phenotype score, six physical fitness items, and posture; 3) psychophysiological markers, including attention and executive function, exercise self-efficacy, and depressive symptoms; 4) biochemical markers, including brain-derived neurotrophic factors; 5) sociological markers, including social networks; and 6) lifestyle markers, including energy intake, step counts, and sleep quality. The detailed measurement of secondary outcomes, adherence, and adverse events are shown in eMaterial 3.

A baseline assessment was conducted 2 weeks before the commencement of the intervention, and a follow-up assessment was conducted within 1 week after the intervention. All outcomes and methods were reported in detail in our previously published protocol paper^10^ and were assessed appropriately according to a pre-specified protocol. The results of the posture markers will be reported separately as secondary analysis results.

### Sample size

As the previous phase 2 trial showed that the home-based Radio-Taiso provided a moderate effect size (Cohen’s d = 0.395) on the Mental Component Summary scores,^12^ the sample size was calculated based on the effect size, 5% alpha error, and 80% power, resulting in a total of 204. The dropout rate was estimated at 10%; thus, the target sample size was 226.^10^

### Statistical analysis

All analyses were performed using R version 4.1.2 (The R Foundation for Statistical Computing, Vienna, Austria) in compliance with a previously described statistical analysis plan.^10^ Continuous variables at baseline are expressed as mean (standard deviation) or median (interquartile range [IQR]), while categorical variables are presented as *n* (%). Between-group differences in retention percentage and incidence of adverse events were compared using Fisher’s exact or the chi-square (*χ*^2^) test.

#### Main analysis

The results of the main analysis were interpreted based on the intention-to-treat principle. A general linear model adjusted for baseline values and allocation stratification factors was employed to test between-group differences (control–intervention group) in the change of each outcome to assess the home-based Radio-Taiso’s effectiveness. The differences are indicated as adjusted mean differences and 95% confidence intervals. The effect sizes were evaluated using eta-squared (*η*^2^) (small: *η*^2^ = 0.01, medium: *η*^2^ = 0.06, large: *η*^2^ = 0.14). *η*^2^ was calculated by dividing the sum of sequence_effect_ by sequence_effect_. The main analysis used a full analysis set that excluded participants who 1) were found not to meet the eligibility criteria after randomization, 2) never participated in the intervention program, and 3) had no follow-up data.^22^

#### Additional analysis

Additional analyses using a per-protocol set were performed to assess whether protocol adherence affected the results. This sample included participants who practiced the Radio-Taiso at home for at least 75% of the intervention.^23^ A sensitivity analysis was also performed to assess the attrition bias due to missing values. Missing data were subjected to a multiple imputations (chained equation) method that generated 20 imputed datasets using each outcome at the baseline, stratification, and group factors.

Furthermore, subgroup analyses were conducted to examine the heterogeneity in the effectiveness of the Radio-Taiso on outcomes based on stratification factors (male vs female, <75 vs ≥75 years, and pre-frailty vs frailty) and the interactions between the Radio-Taiso and each stratification factor (effect modification by stratification factors) were tested.

## RESULTS

### Enrollment

**Figure 3.**
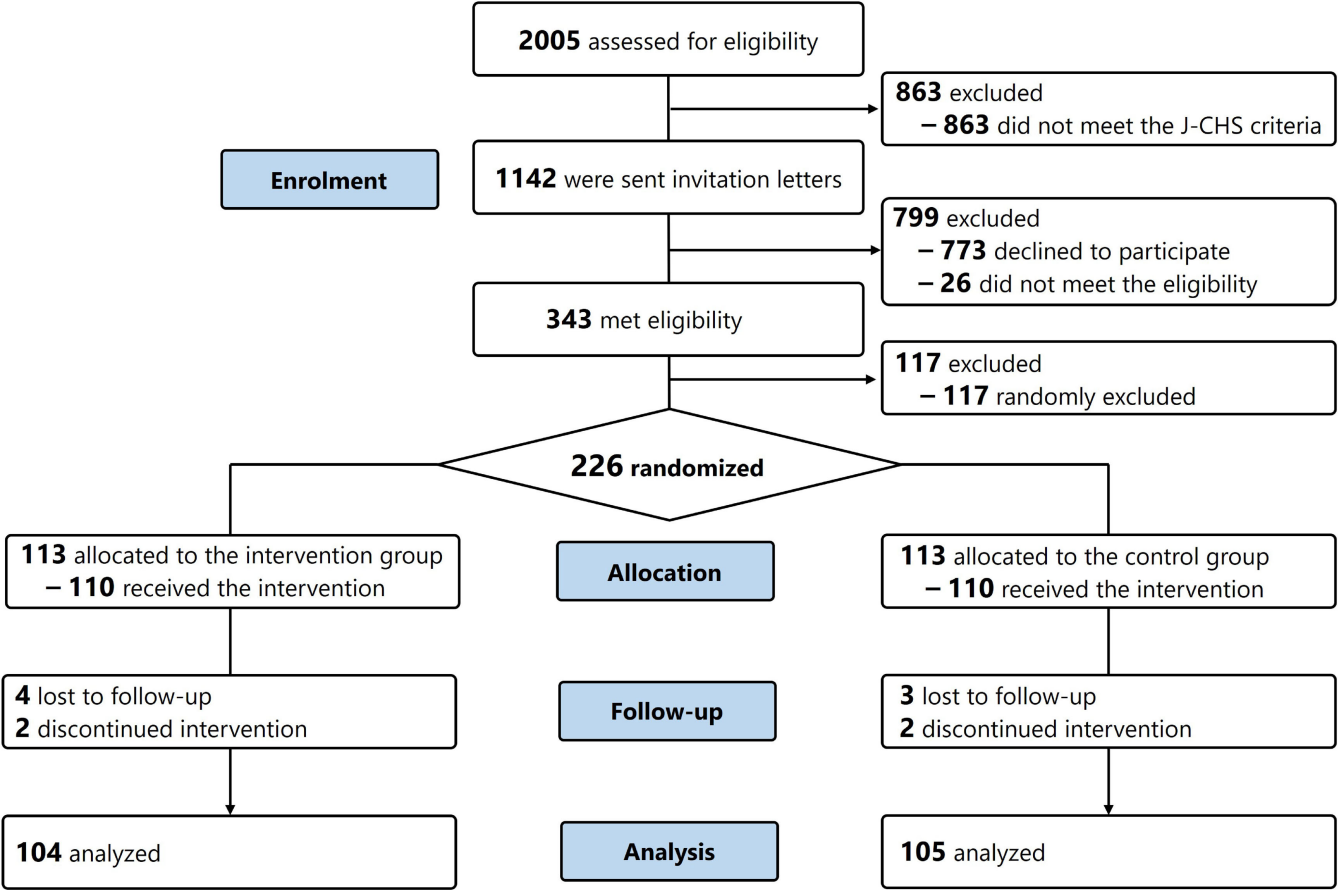
Flow diagram of the trial. J-CHS, Japanese Cardiovascular Health Study

The trial flow from recruitment to analysis is shown in Figure 3. From October 2021 to February 2022, 2,005 community-dwelling older Japanese adults participated in the trial-ready cohorts. Among them, 1,142 who met the criteria for frailty or pre-frailty were sent trial invitations. Eventually, 410 individuals participated in the orientations, and 343 met all eligibility criteria for participation. Among the eligible participants, 226 were randomly selected and invited for a baseline assessment. Ultimately, 220 (97.3%) individuals participated in the baseline assessment and were randomly assigned to the predetermined two groups.

### Baseline characteristics

Table 1 summarizes the characteristics of all participants and each group at baseline. The median age of all participants was 78 (IQR, 74–83) years; 70.0% were women, and 9.5% were frail. The Mental Component Summary, Physical Component Summary, and Role and Social Component Summary scores at the baseline assessment for all participants were 55 (IQR, 48–60), 45 (IQR, 39–51), and 52 (IQR, 42–57), respectively.

**Table 1.** Baseline characteristics of all participants and the allocation groups

	All	Intervention	Control
	*n* = 220	*n* = 110	*n* = 110
Age, years, median [IQR]	78 [74–83]	78 [74–82]	78 [74–82]
Sex, women	154 (70.0)	76 (69.1)	78 (70.9)
Hypertension, yes	99 (45.0)	43 (39.1)	56 (50.9)
Heart disease, yes	31 (14.1)	17 (15.5)	14 (12.7)
Diabetes, yes	24 (10.9)	13 (11.8)	11 (10.0)
Hyperlipidemia, yes	74 (33.6)	36 (32.7)	38 (34.5)
Osteoporosis, yes	38 (17.3)	16 (14.5)	22 (20.0)
Respiratory disease, yes	23 (10.5)	12 (10.9)	11 (10.0)
Low-back pain, yes	94 (42.7)	39 (35.5)	55 (50.0)
Knee pain, yes^a^	62 (28.2)	24 (21.8)	38 (34.5)

Data are shown as *n* (%) unless otherwise noted.

^a^Missing values, *n* = 1.

### Adherence

Overall, 210 participants completed the follow-up assessment (retention percentage, 92.9%), with no significant difference between the two groups (*P* = 1.000). During the follow-up period, three individuals (intervention: *n* = 1, control: *n* = 2) withdrew consent, and seven (intervention: *n* = 4, control: *n* = 3) were lost to follow-up. After excluding one participant who neither attended the instructions class nor practiced at home, 209 individuals (intervention: *n* = 104, control: *n* = 105) were included in the full analysis set.

Adherence to the Radio-Taiso was assessed by analyzing 103 exercise diaries collected from the participants. The median percentage of Radio-Taiso practices and the total number of practices during the intervention period were 94.0% (IQR, 73.2–98.8%) and 163 (IQR, 111–220) times, respectively. Twenty-eight participants did not meet the 75% practice rate in the intervention group, and after excluding these participants, 75 were included in the per-protocol analysis.

### Adverse events

The number of adverse events reported by the participants was 92 and 94 in the intervention and control groups, respectively. Adverse events in the intervention and control groups included 30 and 21 events of pain, 4 and 1 falls, and 58 and 72 other adverse events, respectively. The *χ*^2^ test showed no difference in the incidence of all adverse events between the two groups (intervention: 47.1%, control: 40.0%, *P* = 0.300). Trial physicians determined that three adverse events were possibly related to Radio-Taiso, all of which were mild pain.

### Efficacy outcome

Table 2 shows the mean changes in efficacy outcomes between the intervention and control groups based on the full analysis set. No significant difference was found in the mean changes in the HRQoL summary scores or subscales between the two groups. However, the intervention group showed greater improvement in the 8-foot up-and-go test (0.3 s [*F* (1, 195) = 6.36, *P* = 0.012, *η*^2^ = 0.03]) and the 2-min step-in-place test (3.2 steps [*F* (1, 182) = 4.53, *P* = 0.035, *η*^2^ = 0.02]) than the control group. The Home-Exercise Barriers Self-Efficacy Scale score in the intervention group declined less than that in the control group (1.4 points [*F* (1, 203) = 4.60, *P* = 0.033, *η*^2^ = 0.02]).

**Table 2.** Comparison of the change in the outcomes between the intervention and control arms based on the analysis using full analysis set

	Intervention				Control				Benefits
	*n*	Baseline	Follow-up	Change	*n*	Baseline	Follow-up	Change	Group difference^a^
**Health-related quality of life**									
*MCS, point*	104	55.1 (8.9)	55.9 (8.9)	0.8 (6.7)	105	53.3 (8.1)	54.3 (8.9)	1.0 (6.4)	−0.4 (−2.1 to 1.3)
*PCS, point*	104	45.3 (8.7)	45.0 (9.7)	−0.3 (7.6)	105	42.9 (8.5)	45.0 (10.0)	2.1 (7.2)	1.7 (−0.3 to 3.7)
*RCS, point*	104	47.7 (11.2)	48.5 (11.2)	0.7 (12.9)	105	50.8 (10.0)	49.4 (10.2)	−1.4 (10.5)	−0.2 (−2.9 to 2.5)
*Physical function, point*	104	46.6 (8.2)	46.2 (8.9)	−0.4 (6.6)	105	46.1 (8.0)	46.5 (8.9)	0.5 (6.0)	0.7 (−0.9 to 2.4)
*Physical role, point*	104	47.4 (9.7)	47.7 (9.6)	0.3 (10.4)	105	47.3 (10.0)	48.3 (9.6)	1.0 (8.9)	0.6 (−1.7 to 2.8)
*Body pain, point*	104	45.7 (9.9)	46.9 (11.2)	1.2 (9.6)	105	44.0 (9.9)	46.3 (10.2)	2.3 (8.5)	0.4 (−1.9 to 2.7)
*General health, point*	104	52.5 (8.6)	52.8 (9.0)	0.3 (6.4)	105	49.4 (8.9)	51.0 (9.3)	1.6 (6.8)	0.5 (−1.3 to 2.2)
*Vitality, point*	104	52.8 (9.6)	53.4 (9.7)	0.6 (7.8)	105	52.8 (8.2)	53.2 (9.7)	0.4 (6.8)	−0.2 (−2.1 to 1.7)
*Social function, point*	104	49.5 (10.6)	49.1 (10.7)	−0.4 (10.6)	105	50.6 (9.5)	49.2 (10.4)	−1.4 (9.7)	−0.5 (−2.9 to 1.9)
*Emotional role, point*	104	47.8 (10.5)	49.5 (9.7)	1.8 (11.0)	105	49.3 (9.6)	49.5 (9.0)	0.2 (8.2)	−0.8 (−3.0 to 1.4)
*Mental health, point*	104	54.2 (9.3)	55.5 (8.7)	1.3 (6.9)	105	53.2 (8.9)	54.1 (8.9)	0.9 (6.7)	−0.8 (−2.5 to 0.9)
**Physical parameters**									
*Frailty phenotype, point*	97	1.5 (0.7)	0.8 (0.9)	−0.7 (0.7)	103	1.5 (0.7)	1.0 (0.8)	−0.5 (0.9)	0.2 (0.0 to 0.4)
*8-foot up-and-go, s*	97	6.2 (1.5)	5.7 (1.6)	−0.5 (0.8)	104	6.1 (1.3)	5.9 (1.8)	−0.2 (1.1)	0.3 (0.1 to 0.6)^*^
*Chair stand, times/30 s*	97	16.6 (4.2)	17.1 (4.1)	0.5 (3.2)	103	16.2 (3.8)	16.1 (3.8)	−0.1 (2.8)	−0.7 (−1.5 to 0.0)
*Arm curl, times/30 s*	97	16.7 (4.2)	19.8 (3.8)	3.1 (3.2)	103	16.6 (4.6)	19.1 (4.6)	2.5 (3.5)	−0.6 (−1.5 to 0.2)
*Chair sit-and-reach, cm*	97	8.1 (11.5)	7.2 (11.4)	−0.9 (5.3)	102	6.6 (13.4)	5.3 (13.7)	−1.3 (6.4)	−0.6 (−2.2 to 1.0)
*Back scratch, cm*	96	−7.8 (11.2)	−6.9 (11.0)	0.8 (4.9)	104	−8.5 (12.0)	−8.1 (12.4)	0.4 (4.7)	−0.5 (−1.8 to 0.8)
*2-min step-in-place, times*	88	104.8 (13.2)	112.5 (13.2)	7.7 (10.2)	100	101.6 (15.7)	107.4 (13.7)	5.7 (12.9)	−3.2 (−6.2 to −0.2)^*^
**Psychological parameters**									
*TMT part A, s*	98	60.9 (22.9)	48.0 (16.3)	−12.9 (18.7)	104	61.3 (29.3)	52.6 (25.0)	−8.7 (21.6)	4.4 (0.0 to 8.9)
*TMT part B, s*	98	109.6 (54.6)	116.0 (51.5)	6.4 (41.2)	104	117.0 (59.5)	127.8 (61.5)	10.8 (39.7)	6.6 (−3.7 to 17.0)
*HEBS, score*	104	21.1 (5.2)	20.5 (5.2)	−0.6 (5.2)	105	20.4 (4.2)	18.8 (5.3)	−1.6 (5.0)	−1.4 (−2.6 to −0.1)^*^
*GDS, score*	104	3.6 (2.9)	3.5 (3.0)	−0.1 (1.8)	105	4.3 (3.2)	4.0 (3.1)	−0.3 (2.1)	0.0 (−0.5 to 0.5)
**Biochemical parameters**									
*BDNF, µg/mL*	98	10.2 (4.8)	9.8 (5.3)	−0.4 (5.5)	104	10.0 (5.8)	10.7 (6.8)	0.7 (5.2)	1.0 (−0.4 to 2.4)
**Sociological parameters**									
*LSNS-6, score*	104	14.6 (5.6)	14.5 (5.3)	−0.1 (3.4)	104	14.4 (6.3)	14.0 (5.9)	−0.4 (4.0)	−0.4 (−1.3 to 0.6)
**Habitual lifestyle parameters**									
*Energy intake, kcal/day*	104	1,950 (727)	1,961 (599)	11 (531)	105	2,015 (628)	1,973 (587)	−42 (454)	−29 (−144 to 86)
*Step counts, steps/day*	98	5,424 (2,971)	4,391 (2,691)	−1,033 (1,183)	103	5,566 (2,624)	4,511 (2,191)	−1,055 (1,283)	5 (−290 to 300)
*PSQI, score*	104	6.0 (3.6)	6.0 (3.5)	0.0 (2.0)	103	6.7 (3.5)	6.3 (3.2)	−0.3 (1.9)	−0.3 (−0.8 to 0.2)

BDNF, brain-derived neurotrophic factor; GDS, Geriatric Depression Scale; HEBS, Home-Exercise Barrier Self-Efficacy Scale; LSNS-6, Lubben Social Network Scale-6; MCS, mental component summary; PCS, physical component summary; PSQI, Pittsburgh Sleep Quality Index; RCS, role/social component summary; TMT, trail-making test.

Baseline, follow-up, and change values are shown as mean (standard deviation).

^a^Group differences (the control–intervention groups) in the change of each outcome are shown as mean differences (95% confidence intervals) adjusted for allocation stratification factors and baseline values.

^*^P < 0.05.

Per-protocol analyses showed that the intervention group improved more in the frailty phenotype scores (0.3 points [*F* (1, 169) = 9.53, *P* = 0.002, *η*^2^ = 0.05]), the 8-foot up-and-go test (0.5 s [*F* (1, 171) = 10.88, *P* = 0.001, *η*^2^ = 0.06]), chair stand test (1.0 times [*F* (1, 170) = 6.06, *P* = 0.015, *η*^2^ = 0.03]), and Trail Making Test part A (5.5 s [*F* (1, 171) = 5.16, *P* = 0.024, *η*^2^ = 0.03]) than the control group (Table 3). The Home-Exercise Barriers Self-Efficacy Scale in the intervention group had a smaller decline than that in the control group (2.3 points [*F* (1, 174) = 12.21, *P* = 0.001, *η*^2^ = 0.07]).

**Table 3.** Comparison of the change in the outcomes between the intervention and control arms based on the per-protocol analysis

	Intervention				Control				Benefits
	*n*	Baseline	Follow-up	Change	*n*	Baseline	Follow-up	Change	Group difference^a^
**Health-related quality of life**									
*MCS, point*	75	55.2 (8.4)	56.0 (7.7)	0.8 (6.0)	105	53.3 (8.1)	54.3 (8.9)	1.0 (6.4)	−0.3 (−2.1 to 1.5)
*PCS, point*	75	46.0 (8.6)	45.8 (9.2)	−0.3 (6.9)	105	42.9 (8.5)	45.0 (10.0)	2.1 (7.2)	1.7 (−0.4 to 3.7)
*RCS, point*	75	47.7 (11.6)	49.9 (9.8)	2.3 (12.1)	105	50.8 (10.0)	49.4 (10.2)	−1.4 (10.5)	−1.5 (−4.2 to 1.3)
*Physical function, point*	75	47.4 (7.8)	47.2 (7.7)	−0.2 (6.0)	105	46.1 (8.0)	46.5 (8.9)	0.5 (6.0)	0.4 (−1.3 to 2.1)
*Physical role, point*	75	48.1 (9.7)	49.2 (7.7)	1.1 (9.2)	105	47.3 (10.0)	48.3 (9.6)	1.0 (8.9)	−0.4 (−2.6 to 1.8)
*Body pain, point*	75	45.7 (8.9)	47.8 (10.4)	2.1 (9.6)	105	44.0 (9.9)	46.3 (10.2)	2.3 (8.5)	−0.3 (−2.8 to 2.1)
*General health, point*	75	52.7 (7.6)	53.4 (8.4)	0.7 (5.7)	105	49.4 (8.9)	51.0 (9.3)	1.6 (6.8)	0.2 (−1.7 to 2.0)
*Vitality, point*	75	53.5 (9.3)	53.8 (8.1)	0.2 (7.4)	105	52.8 (8.2)	53.2 (9.7)	0.4 (6.8)	−0.1 (−2.1 to 1.9)
*Social function, point*	75	49.1 (10.6)	50.2 (9.8)	1.1 (9.5)	105	50.6 (9.5)	49.2 (10.4)	−1.4 (9.7)	−1.7 (−4.3 to 0.8)
*Emotional role, point*	75	48.0 (10.6)	51.0 (7.6)	3.0 (10.3)	105	49.3 (9.6)	49.5 (9.0)	0.2 (8.2)	−1.9 (−4.1 to 0.3)
*Mental health, point*	75	54.4 (9.0)	56.2 (8.1)	1.8 (6.2)	105	53.2 (8.9)	54.1 (8.9)	0.9 (6.7)	−1.1 (−2.9 to 0.7)
**Physical parameters**									
*Frailty phenotype, point*	72	1.4 (0.6)	0.6 (0.7)	−0.8 (0.7)	103	1.5 (0.7)	1.0 (0.8)	−0.5 (0.9)	0.3 (0.1 to 0.6)^*^
*8-foot up-and-go, s*	73	6.1 (1.5)	5.5 (1.3)	−0.6 (0.6)	104	6.1 (1.3)	5.9 (1.8)	−0.2 (1.1)	0.5 (0.2 to 0.7)^*^
*Chair stand, times/30 s*	73	16.3 (4.0)	17.2 (3.9)	0.9 (3.1)	103	16.2 (3.8)	16.1 (3.8)	−0.1 (2.8)	−1.0 (−1.8 to −0.2)^*^
*Arm curl, times/30 s*	72	16.5 (4.1)	20.0 (3.7)	3.5 (3.1)	103	16.6 (4.6)	19.1 (4.6)	2.5 (3.5)	−0.9 (−1.8 to 0.0)
*Chair sit-and-reach, cm*	72	8.7 (11.0)	8.0 (10.3)	−0.6 (4.3)	102	6.6 (13.4)	5.3 (13.7)	−1.3 (6.4)	−1.0 (−2.7 to 0.6)
*Back scratch, cm*	71	−6.8 (10.0)	−5.7 (9.9)	1.1 (3.8)	104	−8.5 (12.0)	−8.1 (12.4)	0.4 (4.7)	−0.8 (−2.1 to 0.6)
*2-min step-in-place, times*	67	104.4 (12.8)	111.7 (13.2)	7.3 (10.0)	100	101.6 (15.7)	107.4 (13.7)	5.7 (12.9)	−2.5 (−5.8 to 0.7)
**Psychological parameters**									
*TMT part A, s*	73	58.5 (19.8)	45.4 (12.9)	−13.1 (17.0)	104	61.3 (29.1)	52.6 (25.0)	−8.7 (21.6)	5.5 (0.7 to 10.3)^*^
*TMT part B, s*	73	99.2 (41.5)	107.9 (45.1)	8.8 (33.8)	104	117.1 (59.5)	127.8 (61.5)	10.8 (39.7)	5.2 (−5.6 to 16.1)
*HEBS, score*	75	22.0 (4.2)	21.9 (4.0)	−0.2 (4.4)	105	20.4 (4.2)	18.8 (5.3)	−1.6 (5.0)	−2.3 (−3.6 to −1.0)^*^
*GDS, score*	75	3.5 (2.9)	3.4 (2.8)	−0.2 (1.9)	105	4.3 (3.2)	4.0 (3.1)	−0.3 (2.1)	0.1 (−0.5 to 0.6)
**Biochemical parameters**									
*BDNF, µg/mL*	73	9.9 (5.0)	9.7 (5.3)	−0.2 (5.7)	104	1.0 (0.6)	1.1 (0.7)	0.7 (5.2)	1.0 (−0.5 to 2.5)
**Sociological parameters**									
*LSNS-6, score*	75	14.6 (5.1)	14.7 (5.0)	0.1 (3.6)	104	14.4 (6.3)	14.0 (5.9)	−0.4 (4.0)	−0.5 (−1.6 to 0.5)
**Habitual lifestyle parameters**									
*Energy intake, kcal/day*	75	1,871 (558)	1,913 (524)	−42 (440)	105	2,015 (628)	1,973 (587)	−42 (454)	−31 (−152 to 90)
*Step counts, steps/day*	73	5,446 (2,387)	4,404 (2,048)	−1,042 (1,192)	103	5,566 (2,624)	4,511 (2,191)	−1,055 (1,283)	33 (−280 to 346)
*PSQI, score*	75	6.1 (3.5)	6.2 (3.3)	0.0 (2.1)	103	6.7 (3.5)	6.3 (3.2)	−0.3 (1.9)	−0.2 (−0.8 to 0.3)

BDNF, brain-derived neurotrophic factor; GDS, Geriatric Depression Scale; HEBS, Home-Exercise Barrier Self-Efficacy Scale; LSNS-6, Lubben Social Network Scale-6; MCS, mental component summary; PCS, physical component summary; PSQI, Pittsburgh Sleep Quality Index; RCS, role/social component summary; TMT, trail making test.

Baseline, follow-up, and change values are shown as mean (standard deviation).

^a^Group differences (the control–intervention groups) in the change of each outcome are shown as mean differences (95% confidence intervals) adjusted for allocation stratification factors and baseline values.

^*^P < 0.05.

The statistical significance of the multiple imputed set was consistent with that of the full analysis set for every outcome parameter (see eTable 1). Subgroup analysis based on sex revealed that the effectiveness of Radio-Taiso for chair stand, chair sit-and-reach, and 2-min step-in-place was more pronounced in men than in women. Additionally, the effectiveness of Radio-Taiso for the 2-min step-in-place was more significant in the older age group than in the younger age group. The efficacy of the frailty phenotype score, chair stand, and chair sit-and-reach was higher in the pre-frailty group than in the frailty group. Conversely, the Trail Making Test Part A and the Home-Exercise Barriers Self-Efficacy Scale demonstrated higher efficacy in the frail group than in the pre-frail group (See eTable 2, eTable 3 and eTable 4).

## DISCUSSION

Radio-Taiso is recognized by 97% of Japanese and is implemented in various settings^9^^,^^24^; however, its effectiveness has rarely been documented. To the best of our knowledge, this trial is the first to assess the effectiveness of the home-based Radio-Taiso on the mental domain of HRQoL in older adults with frailty.^10^ This study reconfirmed that Radio-Taiso is an exercise program that can be safely practiced even in unsupervised situations (only three mild pains, 0.01%). Our results showed that while home-based Radio-Taiso benefited agility/balance, aerobic endurance, and exercise self-efficacy, such physical and psychological changes did not improve mental health in older adults with frailty.

The reason our hypothesis was not supported remains unclear. The phase 2 trial was conducted during the early phase of the COVID-19 pandemic (from May to August 2021).^12^ The lifestyle and mental health of older adults with frailty were poor at that time, with severe restrictions on outdoor activities; thus, home-based Radio-Taiso may have functioned as a coping strategy for maintaining mental health during the period.^12^ However, this trial was conducted from June to September 2022, when the vaccine was more widely available, and social and psychological adaptation to COVID-19 may have masked the effectiveness of the home-based Radio-Taiso program.

Few home exercise programs have been established that impact the mental domain positively.^7^ Thus, the optimal duration of exercise interventions to observe favorable changes in mental health outcomes in older adults with frailty remains unclear.^6^ Previous trials have shown that even longer exercise interventions (6–24 months) did not provide favorable changes in mental health outcomes.^25^^,^^26^ Additionally, exercise interventions targeting mental health improvement, including depression, anxiety, and distress, have been reported to be less effective with longer durations than shorter interventions.^27^ This phenomenon is thought to be associated with decreased adherence as intervention durations extend. Future research should identify exercise modalities, including frequency and duration, that can consistently produce positive changes in the mental health of older adults with frailty.

It should be noted that exercise programs that positively impact older adults’ mental domains may be determined by the social support environment surrounding participants, as well as exercise modalities. Group-based exercise programs have been associated with improved socio-psychological outcomes compared to individual exercise, primarily due to social interaction.^28^ Notably, several randomized controlled trials have demonstrated that social support from others involved in exercise positively influences life satisfaction and reduces loneliness in older adults.^29^^,^^30^ However, home-based exercise programs may limit these effects due to limited interaction with others. To observe positive changes in the mental domain with home-based Radio-Taiso, it may be necessary to deliver it in a manner that facilitates interaction and support from others, such as introducing an online video system.

Although the home-based Radio-Taiso program showed a gain of 0.3 s in the 8-foot up-and-go test and two steps in the 2-min step-in-place test, whether these differences mean more than clinically important minimal differences is unclear.^31^ Thus, the clinical benefits of the intervention should be interpreted carefully. Furthermore, the effect size of the difference in the between-group change is small to moderate (*η*^2^ = 0.02–0.03), indicating that the substantial benefit of the intervention on agility/balance and aerobic endurance in older adults with frailty may be mild. We presumed this might be related to the lower intensity (3.7 metabolic equivalents) and shorter exercise duration (3–4 min) of Radio-Taiso than progressive resistance training.^10^^,^^16^^,^^32^

In the per-protocol analysis, home-based Radio-Taiso effectively enhanced frailty phenotype score, lowered body muscle strength, and improved attention function; thus, maintaining adherence to Radio-Taiso could enhance its effectiveness. The intervention group showed a smaller decline in exercise self-efficacy than the control group, which appears to be related to the high feasibility of the Radio-Taiso (practice rate: 94.0%). Adherence to the Radio-Taiso was better than previous home-based exercise programs for older adults with frailty.^33^ This finding suggests that the familiarity and acceptability of the Radio-Taiso motivate participants to sustain exercise, maintaining self-efficacy in daily living.

It is important to acknowledge that our study may exhibit self-selection bias due to volunteers, as participants with a positive inclination towards exercise interventions may have opted to participate. Radio-Taiso is a unique exercise program specific to Japan and relatively unknown in other countries. Therefore, there may be limitations in generalizing the findings of this study to populations with negative feelings toward exercise or to populations outside of Japan. Nevertheless, uniquely standardized exercise programs in different regions and countries through various channels, including schools, workplaces, and communities, may aid in disseminating public health exercises. Disclosing allocation information to participants and exercise instructors increases the risk of performance bias. The results of the secondary outcomes and subgroup analyses (see eTable 1, eTable 2 and eTable 3) should be carefully interpreted, considering the issues associated with multiple testing.

In conclusion, this phase 3 trial supports the findings of our previously published phase 2 trial that home-based Radio-Taiso is a safe and acceptable exercise program for older adults with frailty. Although it provided positive changes in agility/balance, aerobic endurance, and exercise self-efficacy in this population, these changes did not improve their HRQoL.

## SUPPLEMENTARY MATERIAL

eMaterial 1. CONSORT 2010 checklist of information to include when reporting a randomized trial^*^

eMaterial 2. The exclusion criteria of this study

eMaterial 3. Detailed measurement for secondary outcomes, adherence, and adverse events

eTable 1. Consistency of the home-based Radio-Taiso’s effectiveness between the full analysis and multiple imputed sets. ^a^Group differences (the control–intervention groups) in the change of each outcome are shown as mean differences (95% confidence intervals) adjusted for allocation stratification factors and baseline values. ^*^*P* < 0.05. BDNF, brain-derived neurotrophic factor; GDS, Geriatric Depression Scale; HEBS, Home-Exercise Barrier Self-Efficacy Scale; LSNS-6, Lubben Social Network Scale-6; MCS, mental component summary; PCS, physical component summary; PSQI, Pittsburgh Sleep Quality Index; RCS, role/social component summary; TMT, trail making test.

eTable 2. Effect modification of the home-based Radio-Taiso stratified by sex. ^a^Group differences (the control–intervention groups) in the change of each outcome are shown as mean differences (95% confidence intervals) adjusted for allocation stratification factors and baseline values. Effect modification was tested for group × sex interactions. BDNF, brain-derived neurotrophic factor; GDS, Geriatric Depression Scale; HEBS, Home-Exercise Barrier Self-Efficacy Scale; LSNS-6, Lubben Social Network Scale-6; MCS, mental component summary; PCS, physical component summary; PSQI, Pittsburgh Sleep Quality Index; RCS, role/social component summary; TMT, trail making test.

eTable 3. Effect modification of the home-based Radio-Taiso stratified by age category. ^a^Group differences (the control–intervention groups) in the change of each outcome are shown as mean differences (95% confidence intervals) adjusted for allocation stratification factors and baseline values. Effect modification was tested for group × sex interactions. BDNF, brain-derived neurotrophic factor; GDS, Geriatric Depression Scale; HEBS, Home-Exercise Barrier Self-Efficacy Scale; LSNS-6, Lubben Social Network Scale-6; MCS, mental component summary; PCS, physical component summary; PSQI, Pittsburgh Sleep Quality Index; RCS, role/social component summary; TMT, trail making test.

eTable 4. Effect modification of the home-based Radio-Taiso stratified by severity of frailty. ^a^Group differences (the control–intervention groups) in the change of each outcome are shown as mean differences (95% confidence intervals) adjusted for allocation stratification factors and baseline values. Effect modification was tested for group × sex interactions. BDNF, brain-derived neurotrophic factor; GDS, Geriatric Depression Scale; HEBS, Home-Exercise Barrier Self-Efficacy Scale; LSNS-6, Lubben Social Network Scale-6; MCS, mental component summary; PCS, physical component summary; PSQI, Pittsburgh Sleep Quality Index; RCS, role/social component summary; TMT, trail making test.
