# Supplementary material for: Effects of Radio-Taiso on Health-related Quality of Life in Older Adults With Frailty: a Randomized Controlled Trial
Source: J Epidemiol. 2024 Oct 5;34(10):467–76. doi: 10.2188/jea.JE20230317 (PMC11405370; doi:10.2188/jea.JE20230317)
Supplement: Supplementary file 1 [file je-34-467-s001.pdf]

eMaterial 1

CONSORT 2010 checklist of information to include when reporting a randomised trial*

Section/Topic	Item No	Checklist item	Reported on page No
**Title and abstract**			
	1a	Identification as a randomised trial in the title	1
	1b	Structured summary of trial design, methods, results, and conclusions (for specific guidance see CONSORT for abstracts)	4–5
**Introduction**			
Background and objectives	2a	Scientific background and explanation of rationale	6-8
	2b	Specific objectives or hypotheses	8
**Method**			
Trial design	3a	Description of trial design (such as parallel, factorial) including allocation ratio	8
	3b	Important changes to methods after trial commencement (such as eligibility criteria), with reasons	N.A.
Participants	4a	Eligibility criteria for participants	9 and Additional file 2
	4b	Settings and locations where the data were collected	8–9
Interventions	5	The interventions for each group with sufficient details to allow replication, including how and when they were	10–12

		actually administered	
Outcomes	6a	Completely defined pre-specified primary and secondary outcome measures, including how and when they were assessed	12–14 Additional file 3
	6b	Any changes to trial outcomes after the trial commenced, with reasons	N.A.
Sample size	7a	How sample size was determined	14
	7b	When applicable, explanation of any interim analyses and stopping guidelines	N.A.
Randomisation:			
Sequence generation	8a	Method used to generate the random allocation sequence	10
	8b	Type of randomisation; details of any restriction (such as blocking and block size)	10
Allocation concealment mechanism	9	Mechanism used to implement the random allocation sequence (such as sequentially numbered containers), describing any steps taken to conceal the sequence until interventions were assigned	10
Implementation	10	Who generated the random allocation sequence, who enrolled participants, and who assigned participants to interventions	10
Blinding	11a	If done, who was blinded after assignment to interventions (for example, participants, care providers, those assessing outcomes) and how	10
	11b	If relevant, description of the similarity of interventions	10
Statistical methods	12a	Statistical methods used to compare groups for primary and secondary outcomes	14–15

	12b	Methods for additional analyses, such as subgroup analyses and adjusted analyses	15–16
**Results**			
Participant flow (a diagram is strongly recommended)	13a	For each group, the numbers of participants who were randomly assigned, received intended treatment, and were analysed for the primary outcome	16
	13b	For each group, losses and exclusions after randomisation, together with reasons	Figure 3
Recruitment	14a	Dates defining the periods of recruitment and follow-up	9
	14b	Why the trial ended or was stopped	N.A.
Baseline data	15	A table showing baseline demographic and clinical characteristics for each group	16–17 Table 1
Numbers analysed	16	For each group, number of participants (denominator) included in each analysis and whether the analysis was by original assigned groups	Figure 3 17
Outcomes and estimation	17a	For each primary and secondary outcome, results for each group, and the estimated effect size and its precision (such as 95% confidence interval)	18–19 Tables 2–3
	17b	For binary outcomes, presentation of both absolute and relative effect sizes is recommended	N.A.
Ancillary analyses	18	Results of any other analyses performed, including subgroup analyses and adjusted analyses, distinguishing pre-specified from exploratory	19 Additional files 5–7
Harms	19	All important harms or unintended effects in each group (for specific guidance see CONSORT for harms)	18
**Discussion**			

Limitations	20	Trial limitations, addressing sources of potential bias, imprecision, and, if relevant, multiplicity of analyses	23
Generalisability	21	Generalisability (external validity, applicability) of the trial findings	22–23
Interpretation	22	Interpretation consistent with results, balancing benefits and harms, and considering other relevant evidence	20–23
**Other information**			
Registration	23	Registration number and name of trial registry	5
Protocol	24	Where the full trial protocol can be accessed, if available	9
Funding	25	Sources of funding and other support (such as supply of drugs), role of funders	25

*We strongly recommend reading this statement in conjunction with the CONSORT 2010 Explanation and Elaboration for important clarifications on all the items. If relevant, we also recommend reading CONSORT extensions for cluster randomised trials, non-inferiority and equivalence trials, non-pharmacological treatments, herbal interventions, and pragmatic trials. Additional extensions are forthcoming: for those and for up to date references relevant to this checklist, see www.consort-statement.org.

eMaterial 2. The exclusion criteria of this study

1	Participants who had known inability to participate in at least one of the baseline and follow-up assessments
2	Those diagnosed with dementia or on anti-dementia medication
3	Those who cannot eat, toilet, dress, move, or bath independently
4	Those who were prohibited from exercising by a family physician (except for light-intensity exercise)
5	Those with unstable or severe medical condition that could preclude study participation
6	Those who had angina, myocardial infarction, or cardiac surgery within the past three months
7	Those who practiced Radio-Taiso for more than 1 day/week in the past month
8	Those who participated in specific rehabilitation programs
9	Those who cannot walk independently for more than 10 min
10	Those who participated or planned to participate in other clinical trials
11	Those who had no television at their home
12	Those who could not communicate in Japanese
13	Those judged as ineligible by the principal investigator and trial physicians
14	Those who did not provide consent to participate

eMaterial 3. Detailed measurement for secondary outcomes, adherence, and adverse events

Physical parameters

The frailty phenotype was assessed by summing the five limitations (slowness, weakness, exhaustion, inactivity, and weight loss) using the revised Japanese version of the Cardiovascular Health Study criteria.[1, 2]

The Senior Fitness Test Battery assesses six physical fitness domains: agility/dynamic balance, lower and upper body strength, flexibility, and aerobic endurance.[3] Agility/dynamic balance was assessed by standing up from a chair, walking around a cone 8 feet away as fast as possible, and measuring the time it took to sit down again (8-foot up-and-go test). Lower body muscle strength was assessed by counting the number of times the participants stood up from a chair for 30 seconds (chair stand test). Upper body muscle strength was assessed by counting the number of times the participants flexed and extended their elbows while holding a dumbbell in their dominant hands for 30 seconds (arm curl test). Lower body flexibility was assessed by measuring the distance between the toes of the dominant leg and the fingertips of both hands while sitting on a chair and bending the upper body (chair sit-and-reach test).

Upper-body flexibility was assessed by measuring the distance between the middle fingers when any hand approached diagonally above backward and diagonally below backward (back scratch test). Aerobic endurance was assessed by counting the time the right leg was raised while marching in place for 2 minutes (2-min step-in-place test).

Psychological parameters

Attention and executive function were assessed by measuring the time required for each task in Parts A and B of the Trail Making Test.[4] Exercise self-efficacy was assessed using the Home Exercise Barriers Self-Efficacy Scale.[5] The scale ranges from 6–30 points, with higher scores indicating greater confidence in exercising at home. Depressive symptoms were assessed using the short version of the Geriatric Depression Scale.[6] This scale is rated from 0–15, with higher scores indicating a more depressive mood.

Biochemical parameters

Exercise-induced brain-derived neurotrophic factors may explain improvements in mental health by mediating neuronal differentiation, growth, synaptogenesis, and plasticity.[7] Participants were instructed to fast for at least 2 hours, and their blood

samples were collected from the anterior elbow vein. Plasma brain-derived neurotrophic factor concentrations were assessed using a commercially available two-site sandwich enzyme-linked immunosorbent assay kit (R&D Systems, Minneapolis, MN, USA).

Sociological parameters

Social networks were assessed using the Japanese version of the Lubben Social Network Scale.[8] This scale is rated on a 0–30 point scale, with higher scores indicating a more extensive social network.

Lifestyle parameters

A brief-type self-administered dietary history questionnaire assessed daily energy intake by recalling the average dietary habits over the past month and recording the frequency of each food item consumed.[9, 10] Daily step counts were assessed using a 3-axis accelerometer (Active style Pro HJA-750C; Omron Healthcare, Tokyo, Japan) to indicate daily physical activity level.[11, 12] Participants were instructed to wear the device on their hip during all daily activities except for water activities from waking to bedtime for 7 days from the baseline and after the follow-up assessments. Daily step counts were calculated for samples in which valid records were collected for at least 3 days.[13] Sleep

quality was assessed using the Japanese version of the Pittsburgh Sleep Quality Index,[14, 15] and the total scores on this scale range from 0 to 21, with higher scores indicating poorer daily sleep quality.

Adherence

The retention percentage during the intervention period (percentage of participants who completed the follow-up assessments), practice percentage in participants' homes (number of days that the Radio-Taiso was practiced at least once per day/84 days), and the total number of practices during the intervention period were assessed.

Adverse events

Once every 2 weeks, research assistants recorded whether participants experienced an adverse event, defined as *"any undesirable/unintended sign, symptom, or disease occurring during the intervention, regardless of causality,"* via telephone or face-to-face interviews.[16] The total number of adverse events occurring during the intervention period was recorded, and trial physicians evaluated the severity and causality of the individual adverse events and their association with the Radio-Taiso. The number of adverse events and incidence of all adverse events were assessed.

REFERENCES

1. Fried LP, Tangen CM, Walston J, et al. Frailty in older adults: evidence for a phenotype. J Gerontol A Biol Sci Med Sci. 2001;56:M146-56.

2. Satake S, Arai H. The revised Japanese version of the Cardiovascular Health Study criteria (revised J-CHS criteria). Geriatr Gerontol Int. 2020;20:992-3.

3. Rikli RE, Jones CJ. Development and validation of a functional fitness test for community-residing older adults. J Aging Phys Act. 1999;7:129-61.

4. Army Individual Test Battery. Washington, DC: War Department. Adjutant General's Office; 1944.

5. Arita N, Takenaka K, Shimazaki T. Development of a home-exercise barrier self-efficacy scale for elderly people requiring support and care. J Jpn Phys Ther Assoc. 2014;41:338-46.

6. Yatomi N. The factor structure and item characteristic of the GDS (Geratric Depression Scale) short version in a Japanese elderly sample. Japanese Journal of Gerontology. 1994;16:29-36.

7. Knaepen K, Goekint M, Heyman EM, Meeusen R. Neuroplasticity - exercise-induced response of peripheral brain-derived neurotrophic factor: a systematic review of experimental studies in human subjects. Sports Med. 2010;40:765-

801.

8. Kurimoto A, Awata S, Ohkubo T, et al. [Reliability and validity of the Japanese version of the abbreviated Lubben Social Network Scale]. Nihon Ronen Igakkai Zasshi. 2011;48:149-57.

9. Kobayashi S, Murakami K, Sasaki S, et al. Comparison of relative validity of food group intakes estimated by comprehensive and brief-type self-administered diet history questionnaires against 16 d dietary records in Japanese adults. Public Health Nutr. 2011;14:1200-11.

10. Kobayashi S, Honda S, Murakami K, et al. Both comprehensive and brief self-administered diet history questionnaires satisfactorily rank nutrient intakes in Japanese adults. J Epidemiol. 2012;22:151-9.

11. Oshima Y, Kawaguchi K, Tanaka S, et al. Classifying household and locomotive activities using a triaxial accelerometer. Gait Posture. 2010;31:370-4.

12. Ohkawara K, Oshima Y, Hikihara Y, Ishikawa-Takata K, Tabata I, Tanaka S. Real-time estimation of daily physical activity intensity by a triaxial accelerometer and a gravity-removal classification algorithm. Br J Nutr. 2011;105:1681-91.

13. Troiano RP, Berrigan D, Dodd KW, Mâsse LC, Tilert T, McDowell M. Physical

activity in the United States measured by accelerometer. Med Sci Sports Exerc. 2008;40:181-8.

14. Doi Y, Minowa M, Uchiyama M, et al. Psychometric assessment of subjective sleep quality using the Japanese version of the Pittsburgh Sleep Quality Index (PSQI-J) in psychiatric disordered and control subjects. Psychiatry Res. 2000;97:165-72.

15. Doi Y, Minowa M, Okawa M, Uchiyama M. Development of the Japanese version of the Pittsburgh Sleep Quality Index. Jpn J Psychiatry Treat. 1998;13:755-63 (in Japanese).

16. Osuka Y, Kojima N, Sugie M, et al. Effects of a home-based Radio-Taiso exercise programme on health-related quality of life in older adults with frailty: protocol for an assessor-blind randomised controlled trial. BMJ Open. 2022;12:e063201.

eTable 1. Consistency of the home-based Radio-Taiso's effectiveness between the full analysis and multiple imputed sets

	Full analysis set		Multiple imputed set	
	n	Group difference[a]	n	Group difference[a]
**Health-related quality of life**				
MCS, point	209	-0.4 (-2.1 to 1.3)	220	-0.3 (-2.1 to 1.4)
PCS, point	209	1.7 (-0.3 to 3.7)	220	1.6 (-0.3 to 3.6)
RCS, point	209	-0.2 (-2.9 to 2.5)	220	-0.1 (-2.8 to 2.5)
Physical function, point	209	0.7 (-0.9 to 2.4)	220	0.7 (-0.9 to 2.4)
Physical role, point	209	0.6 (-1.7 to 2.8)	220	0.5 (-1.7 to 2.7)
Body pain, point	209	0.4 (-1.9 to 2.7)	220	0.2 (-2.0 to 2.5)
General health, point	209	0.5 (-1.3 to 2.2)	220	0.3 (-1.5 to 2.0)
Vitality, point	209	-0.2 (-2.1 to 1.7)	220	-0.2 (-2.2 to 1.7)
Social function, point	209	-0.5 (-2.9 to 1.9)	220	-0.6 (-3.2 to 1.9)
Emotional role, point	209	-0.8 (-3.0 to 1.4)	220	-0.8 (-3.0 to 1.3)
Mental health, point	209	-0.8 (-2.5 to 0.9)	220	-0.6 (-2.3 to 1.0)
**Physical parameters**				
Frailty phenotype, point	200	0.2 (0 to 0.4)	220	0.2 (-0.1 to 0.4)
8-foot up-and-go, s	201	0.3 (0.1 to 0.6)*	220	0.3 (0.1 to 0.6)*
Chair stand, times/30s	200	-0.7 (-1.5 to 0.0)	220	-0.7 (-1.5 to 0.1)

Arm curl, times/30s	200	-0.6 (-1.5 to 0.2)	220	-0.6 (-1.4 to 0.2)
Chair sit-and-reach, cm	199	-0.6 (-2.2 to 1.0)	220	-0.6 (-2.2 to 1.1)
Back scratch, cm	200	-0.5 (-1.8 to 0.8)	220	-0.6 (-1.9 to 0.8)
2-min step-in-place, times	188	-3.2 (-6.1 to -0.2)*	220	-3.1 (-6.2 to 0.0)*
**Psychological parameters**				
TMT part A, s	202	4.4 (0 to 8.9)	220	4.4 (-0.2 to 8.9)
TMT part B, s	202	6.6 (-3.7 to 17.0)	220	6.4 (-4.1 to 16.9)
HEBS, score	209	-1.4 (-2.6 to -0.1)*	220	-1.4 (-2.6 to -0.1)*
GDS, score	209	0.0 (-0.5 to 0.5)	220	0.0 (-0.5 to 0.5)
**Biochemical parameters**				
BDNF, μg/mL	202	1.0 (-0.4 to 2.4)	220	0.9 (-0.5 to 2.4)
**Sociological parameters**				
LSNS-6, score	208	-0.4 (-1.3 to 0.6)	220	-0.3 (-1.2 to 0.6)
**Habitual lifestyle parameters**				
Energy intake, kcal/day	209	-29 (-144 to 86)	220	-23 (-139 to 93)
Step counts, steps/day	201	5 (-290 to 300)	220	-41 (-328 to 245)
PSQI, score	207	-0.3 (-0.8 to 0.2)	220	-0.3 (-0.8 to 0.2)

BDNF, brain-derived neurotrophic factor; GDS, Geriatric Depression Scale; HEBS, Home-Exercise Barrier Self-Efficacy Scale; LSNS-6, Lubben Social Network Scale-6; MCS, mental component summary; PCS, physical component summary; PSQI Pittsburgh Sleep Quality Index; RCS, role/social component

summary; TMT, trail making test.

[a]Group differences (the control–intervention groups) in the change of each outcome are show as mean differences (95% confidence intervals) adjusted for allocation stratification factors and baseline values.

[*]$P<0.05$.

eTable 2. Effect modification of the home-based Radio-Taiso stratified by sex

	Male		Female		*P* values for
	n	Group differences[a]	n	Group differences[a]	effect modification
**Health-related quality of life**					
MCS, point	63	-1.2 (-5.0 to 2.7)	146	-0.5 (-2.4 to 1.4)	0.797
PCS, point	63	2.8 (-1.1 to 6.6)	146	1.3 (-1.1 to 3.6)	0.442
RCS, point	63	-0.3 (-4.6 to 4.1)	146	-0.3 (-3.7 to -3.1)	0.794
Physical function, point	63	2.6 (-0.5 to 5.6)	146	-0.1 (-2.1 to 2.0)	0.148
Physical role, point	63	0.8 (-2.7 to 4.2)	146	0.5 (-2.4 to 3.4)	0.895
Body pain, point	63	1.0 (-3.3 to 5.4)	146	-0.1 (-2.7 to 2.6)	0.530
General health, point	63	-0.3 (-4.0 to 3.3)	146	0.6 (-1.4 to 2.6)	0.846
Vitality, point	63	-0.5 (-4.6 to 3.6)	146	-0.3 (-2.5 to 1.9)	0.956
Social function, point	63	-1.0 (-4.3 to 2.4)	146	-0.3 (-3.5 to 2.8)	0.796
Emotional role, point	63	-0.6 (-4.0 to 2.9)	146	-0.9 (-3.7 to 1.9)	0.813
Mental health, point	63	-0.4 (-3.7 to 3.0)	146	-1.4 (-3.4 to 0.6)	0.286
**Physical parameters**					
Frailty phenotype, point	59	0.1 (-0.3 to 0.4)	141	0.2 (0.0 to 0.5)	0.582
8-foot up-and-go, s	58	0.7 (0.0 to 1.3)	143	0.2 (-0.1 to 0.4)	0.110
Chair stand, times/30s	57	-2.4 (-4.2 to -0.6)	143	-0.1 (-0.8 to 0.7)	0.006

Arm curl, times/30s	58	-1.2 (-2.8 to 0.5)	142	-0.4 (-1.4 to 0.6)	0.454
Chair sit-and-reach, cm	57	-4.4 (-7.6 to -1.2)	142	0.9 (-0.9 to 2.7)	0.004
Back scratch, cm	58	0.1 (-2.3 to 2.5)	142	-0.7 (-2.2 to 0.9)	0.700
2-min step-in-place, times	53	-8.8 (-15.2 to -2.3)	135	-1.0 (-4.3 to 2.3)	0.020
**Psychological parameters**					
TMT part A, s	59	4.8 (-4.0 to 13.6)	143	4.6 (-0.6 to 9.9)	0.959
TMT part B, s	59	14.2 (-5.7 to 34.1)	143	3.5 (-8.8 to 15.9)	0.369
HEBS, score	63	-1.3 (-3.9 to 1.2)	146	-1.5 (-2.9 to 0.0)	0.794
GDS, score	63	-0.3 (-1.1 to 0.5)	146	0.1 (-0.5 to 0.8)	0.666
**Biochemical parameters**					
BDNF, µg/mL	59	0.2 (-1.9 to 2.5)	143	1.4 (-0.4 to 3.1)	0.440
Sociological parameters					
LSNS-6, score	63	-0.3 (-2.5 to 1.8)	145	-0.3 (-1.3 to 0.7)	0.970
**Habitual lifestyle parameters**					
Energy intake, kcal/day	63	-36 (-253 to 182)	146	-19 (-155 to 118)	0.923
Step counts, steps/day	58	404 (-69 to 878)	143	-94 (-461 to 273)	0.259
PSQI, score	63	-0.1 (-1.2 to 0.9)	144	-0.3 (-0.9 to 0.3)	0.893

BDNF, brain-derived neurotrophic factor; GDS, Geriatric Depression Scale; HEBS, Home-Exercise Barrier Self-Efficacy Scale; LSNS-6, Lubben Social Network Scale-6; MCS, mental component summary; PCS, physical component summary; PSQI Pittsburgh Sleep Quality Index; RCS, role/social component

summary; TMT, trail making test.

[a]Group differences (the control–intervention groups) in the change of each outcome are show as mean differences (95% confidence intervals) adjusted for allocation stratification factors and baseline values. Effect modification was tested for group × sex interactions.

eTable 3. Effect modification of the home-based Radio-Taiso stratified by age category

	n	Group differences[a]	n	Group differences[a]	*P* values for effect modification
	<75 years		≥75 years		
**Health-related quality of life**					
MCS, point	56	-0.3 (-3.5 to 2.8)	153	-0.4 (-2.5 to 1.6)	0.885
PCS, point	56	2.7 (-1.2 to 6.6)	153	1.3 (-1.1 to 3.6)	0.476
RCS, point	56	-1.0 (-5.0 to 3.0)	153	0.0 (-3.5 to 3.4)	0.854
Physical function, point	56	1.4 (-1.3 to 4.0)	153	0.5 (-1.6 to 2.6)	0.618
Physical role, point	56	0.6 (-2.6 to 3.9)	153	0.6 (-2.3 to 3.4)	0.995
Body pain, point	56	0.3 (-4.1 to 4.7)	153	0.3 (-2.4 to 2.9)	0.870
General health, point	56	0.8 (-3.0 to 4.6)	153	0.3 (-1.7 to 2.3)	0.726
Vitality, point	56	-0.6 (-4.4 to 3.3)	153	-0.1 (-2.3 to 2.2)	0.860
Social function, point	56	0.2 (-3.8 to 4.2)	153	-0.9 (-3.9 to 2.2)	0.649
Emotional role, point	56	-1.8 (-4.6 to 1.0)	153	-0.4 (-3.2 to 2.4)	0.563
Mental health, point	56	-1.4 (-4.0 to 1.2)	153	-0.5 (-2.6 to 1.6)	0.699
**Physical parameters**					
Frailty phenotype, point	53	0.1 (-0.3 to 0.5)	147	0.2 (0.0 to 0.5)	0.646
8-foot up-and-go, s	54	0.1 (-0.1 to 0.3)	147	0.4 (0.1 to 0.7)	0.418
Chair stand, times/30s	54	-0.4 (-1.7 to 0.9)	146	-0.9 (-1.8 to 0.1)	0.582

Arm curl, times/30s	54	0.5 (-0.8 to 1.9)	146	-1.1 (-2.1 to 0.0)	0.097
Chair sit-and-reach, cm	53	0.6 (-2.3 to 3.5)	146	-1.0 (-2.9 to 0.9)	0.372
Back scratch, cm	54	0.3 (-2.4 to 2.9)	146	-0.8 (-2.3 to 0.8)	0.528
2-min step-in-place, times	52	3.3 (-0.7 to 7.3)	136	-5.7 (-9.5 to -1.9)	0.007
**Psychological parameters**					
TMT part A, s	54	4.0 (-1.3 to 9.3)	148	4.6 (-1.3 to 10.4)	0.939
TMT part B, s	54	11.5 (-3.8 to 26.8)	148	4.7 (-8.2 to 17.6)	0.531
HEBS, score	56	-2.3 (-5.0 to 0.5)	153	-1.1 (-2.5 to 0.3)	0.551
GDS, score	56	-0.2 (-1.1 to 0.7)	153	0.1 (-0.5 to 0.7)	0.573
**Biochemical parameters**					
BDNF, μg/mL	54	1.8 (-1.4 to 5.1)	148	0.7 (-0.9 to 2.2)	0.464
Sociological parameters					
LSNS-6, score	55	-0.6 (-2.2 to 1.0)	153	-0.2 (-1.4 to 0.9)	0.649
**Habitual lifestyle parameters**					
Energy intake, kcal/day	56	-12.9 (-258 to 232)	153	-23 (-154 to 108)	0.977
Step counts, steps/day	54	511 (-206 to 1229)	147	-152 (-450 to 147)	0.082
PSQI, score	56	-0.6 (-1.5 to 0.4)	151	-0.2 (-0.8 to 0.4)	0.539

BDNF, brain-derived neurotrophic factor; GDS, Geriatric Depression Scale; HEBS, Home-Exercise Barrier Self-Efficacy Scale; LSNS-6, Lubben Social Network Scale-6; MCS, mental component summary; PCS, physical component summary; PSQI Pittsburgh Sleep Quality Index; RCS, role/social component

summary; TMT, trail making test.

[a]Group differences (the control–intervention groups) in the change of each outcome are show as mean differences (95% confidence intervals) adjusted for allocation stratification factors and baseline values. Effect modification was tested for group × sex interactions.

eTable 4. Effect modification of the home-based Radio-Taiso stratified by severity of frailty

	Pre-frailty		Frailty		*P* values for
	n	Group differences[a]	n	Group differences[a]	effect modification
**Health-related quality of life**					
MCS, point	189	-0.7 (-2.5 to 1.1)	20	2.9 (-3.4 to 9.2)	0.214
PCS, point	189	1.1 (-1.0 to 3.1)	20	8.4 (1.6 to 15.2)	0.026
RCS, point	189	0.1 (-2.6 to 2.8)	20	-4.7 (-17.0 to 7.7)	0.263
Physical function, point	189	0.4 (-1.3 to 2.1)	20	4.5 (-3.6 to 12.7)	0.171
Physical role, point	189	0.4 (-1.8 to 2.7)	20	2.0 (-7.8 to 11.7)	0.699
Body pain, point	189	-0.5 (-2.9 to 1.9)	20	9.0 (3.1 to 15.0)	0.013
General health, point	189	0.1 (-1,8 to 1.9)	20	4.0 (-1.4 to 9.5)	0.185
Vitality, point	189	-0.6 (-2.6 to 1.5)	20	3.0 (-3.5 to 9.5)	0.262
Social function, point	189	-0.6 (-3.0 to 1.9)	20	1.3 (-9.2 to 11.8)	0.953
Emotional role, point	189	-0.8 (-3.0 to 1.4)	20	-2.1 (-13.0 to 8.7)	0.830
Mental health, point	189	-0.9 (-2.6 to 0.9)	20	0.0 (-7.5 to 7.6)	0.722
**Physical parameters**					
Frailty phenotype, point	182	0.3 (0.0 to 0.5)	18	-0.6 (-1.4 to 0.2)	0.011
8-foot up-and-go, s	184	0.3 (0.1 to 0.5)	17	-0.3 (-2.7 to 2.2)	0.775
Chair stand, times/30s	183	-1.0 (-1.8 to -0.2)	17	2.3 (-0.3 to 4.9)	0.015

Arm curl, times/30s	182	-0.8 (-1.7 to 0.1)	18	0.9 (-2.8 to 4.6)	0.272
Chair sit-and-reach, cm	181	-1.1 (-2.7 to 0.5)	18	4.6 (-2.9 to 12.1)	0.048
Back scratch, cm	182	-0.8 (-2.2 to 0.6)	18	2.5 (-2.4 to 7.5)	0.186
2-min step-in-place, times	173	-2.9 (-6.0 to 0.1)	15	-4.6 (-21.0 to 11.7)	0.483
**Psychological parameters**					
TMT part A, s	184	2.9 (-1.5 to 7.2)	18	23.4 (-2.2 to 48.9)	0.023
TMT part B, s	184	7.4 (-3.5 to 18.4)	18	-5.2 (-29.4 to 19.0)	0.696
HEBS, score	189	-2.0 (-3.2 to -0.7)	20	3.8 (-1.4 to 9.1)	0.006
GDS, score	189	0.1 (-0.5 to 0.6)	20	-0.1 (-1.8 to 1.6)	0.693
**Biochemical parameters**					
BDNF, μg/mL	184	0.9 (-0.6 to 2.4)	18	2.5 (-1.9 to 6.9)	0.492
Sociological parameters					
LSNS-6, score	188	-0.1 (-1.0 to 0.9)	20	-3.0 (-6.7 to 0.8)	0.057
**Habitual lifestyle parameters**					
Energy intake, kcal/day	189	-40 (-162 to 82)	20	112 (-250 to 473)	0.519
Step counts, steps/day	184	-57 (-368 to 255)	17	541 (-87 to 1169)	0.193
PSQI, score	187	-0.2 (-0.8 to 0.3)	20	-0.4 (-1.6 to 0.9)	0.605

BDNF, brain-derived neurotrophic factor; GDS, Geriatric Depression Scale; HEBS, Home-Exercise

Barrier Self-Efficacy Scale; LSNS-6, Lubben Social Network Scale-6; MCS, mental component summary; PCS, physical component summary; PSQI Pittsburgh Sleep Quality Index; RCS, role/social component summary; TMT, trail making test.

[a]Group differences (the control–intervention groups) in the change of each outcome are show as mean differences (95% confidence intervals) adjusted for allocation stratification factors and baseline values. Effect modification was tested for group × sex interactions.
